# Accurate Detection of Alzheimer’s Disease Using Lightweight Deep Learning Model on MRI Data

**DOI:** 10.3390/diagnostics13071216

**Published:** 2023-03-23

**Authors:** Ahmed A. Abd El-Latif, Samia Allaoua Chelloug, Maali Alabdulhafith, Mohamed Hammad

**Affiliations:** 1 EIAS Data Science Lab, College of Computer and Information Sciences, Prince Sultan University, P.O. Box 66833, Riyadh 11586, Saudi Arabia; aabdellatif@psu.edu.sa; 2 Department of Mathematics and Computer Science, Faculty of Science, Menoufia University, Shibin El Kom 32511, Egypt; 3 Department of Information Technology, College of Computer and Information Sciences, Princess Nourah bint Abdulrahman University, P.O. Box 84428, Riyadh 11671, Saudi Arabia; mialabdulhafith@pnu.edu.sa; 4 Department of Information Technology, Faculty of Computers and Information, Menoufia University, Shibin El Kom 32511, Egypt

**Keywords:** Alzheimer’s disease, deep learning, detection, Kaggle dataset, lightweight model, MRI data

## Abstract

Alzheimer’s disease (AD) is a neurodegenerative disorder characterized by cognitive impairment and aberrant protein deposition in the brain. Therefore, the early detection of AD is crucial for the development of effective treatments and interventions, as the disease is more responsive to treatment in its early stages. It is worth mentioning that deep learning techniques have been successfully applied in recent years to a wide range of medical imaging tasks, including the detection of AD. These techniques have the ability to automatically learn and extract features from large datasets, making them well suited for the analysis of complex medical images. In this paper, we propose an improved lightweight deep learning model for the accurate detection of AD from magnetic resonance imaging (MRI) images. Our proposed model achieves high detection performance without the need for deeper layers and eliminates the use of traditional methods such as feature extraction and classification by combining them all into one stage. Furthermore, our proposed method consists of only seven layers, making the system less complex than other previous deep models and less time-consuming to process. We evaluate our proposed model using a publicly available Kaggle dataset, which contains a large number of records in a small dataset size of only 36 Megabytes. Our model achieved an overall accuracy of 99.22% for binary classification and 95.93% for multi-classification tasks, which outperformed other previous models. Our study is the first to combine all methods used in the publicly available Kaggle dataset for AD detection, enabling researchers to work on a dataset with new challenges. Our findings show the effectiveness of our lightweight deep learning framework to achieve high accuracy in the classification of AD.

## 1. Introduction

Alzheimer’s disease (AD) is a neurodegenerative condition characterized by cognitive impairment and aberrant protein buildup in the brain. AD is a devastating disease that affects millions of people around the world [1]. It is the most prevalent kind of dementia among the elderly and is characterized by a steady decline in memory, cognition, and the ability to perform daily activities [2]. As the disease progresses, individuals with AD may experience changes in their behavior, mood, and personality, and they may eventually lose the ability to communicate and interact with others. The impact of AD on individuals and their families is significant [3]. The progression of the disease can be emotionally and financially taxing for caregivers, and it can also have a major impact on the overall quality of life for both the individual with AD and their loved ones. Dementia with AD is typically divided into the following stages [4]:

- Early or Preclinical stage: During this stage, the individual may have no symptoms or only mild memory problems.
- Mild Cognitive Impairment (MCI): In this stage, the individual may experience more noticeable memory problems but still has the ability to perform daily activities independently.
- Mild Dementia: During this stage, the individual may have trouble remembering recent events, completing familiar tasks, and communicating effectively. They may also experience confusion, disorientation, and mood swings.
- Moderate Dementia: In this stage, the individual may require assistance with daily activities and have difficulty recognizing friends and family members. They may also experience more severe memory loss, confusion, and changes in personality and behavior.
- Severe Dementia: During this stage, the individual is typically completely dependent on others for their care and may lose the ability to communicate and recognize loved ones.

The early detection of AD is crucial for the development of effective treatments and interventions, as it is more responsive to treatment in its early stages. Magnetic resonance imaging (MRI) is a popular imaging technique for diagnosing and treating AD [5]. MRI allows for the visualization of brain structure and the detection of structural changes that may occur in AD, such as the shrinkage of certain brain regions, changes in brain tissue density, and the accumulation of certain substances in the brain [6]. MRI can also be used to distinguish AD from other causes of dementia and to track the progression of the disease over time. In combination with other diagnostic tools, such as cognitive and neurological assessments, MRI can help support a diagnosis of AD.

There is currently no cure for AD, but there are a number of treatments and interventions that can help manage the symptoms of the disease [7]. These can include medications to improve cognitive function, therapies to address behavioral and psychological symptoms, and support for caregivers. Research is ongoing to develop more effective treatments and to better understand the underlying causes of AD [8,9,10,11,12,13,14,15,16,17,18,19,20,21,22,23]. In recent years, deep learning techniques have been successfully applied to a wide range of medical imaging tasks [24,25,26,27,28], including the detection of AD [8,9,10,11,12,13,14,15,16]. These techniques have the ability to automatically learn and extract features from large datasets, making them well suited for the analysis of complex medical images. However, the use of deep learning for AD detection is not without limitations. The performance of deep learning models can be affected by factors such as the quality and size of the dataset, the choice of model architecture, and the optimization of model parameters.

Despite these limitations, deep learning has shown great potential for the detection of AD, and further research is needed to fully understand its capabilities and limitations in this area. This paper aims to review the recent literature on the use of deep learning for AD detection and to discuss the limitations of related work in this field. In addition, we overcome these limitations by proposing a new lightweight deep learning model for the accurate detection of AD from MRI images. To ensure the robustness and effectiveness of our model, we trained it using a combination of different techniques, including data augmentation, deep learning, and early stopping. We also evaluated the performance of our model using multiple metrics, including accuracy, precision, recall, and F1-score, to provide a comprehensive analysis of its effectiveness. The main novelties of this paper are the following:

- We propose a novel lightweight deep learning model using MRI images for the accurate detection of AD. The proposed model can be employed in real-time applications with high detection performance, unlike other previous models that needed deeper layers to obtain high detection accuracies.
- The proposed model is an end-to-end model that eliminates the use of traditional methods such as feature extraction and classification and combines them all into one stage. In addition, the proposed method consists only of seven layers, which makes the system less complex than other previous deep models and less time-consuming to process.
- We introduce a model that works on binary classification and multi-classification tasks with higher performance than other previous models. Consequently, the proposed model is more robust than earlier deep learning techniques.
- This is the first study that reviews and combines all methods used in such datasets from Kaggle. This paper combines all previous methods that work on the same publicly available Kaggle dataset [29], allowing researchers to work on a dataset with new challenges. Unlike other previous methods that used other unpublished large-size datasets, which are approximately 1.5 Gigabytes with a small number of records, we employed a small-size dataset (only 36 Megabytes) with a large number of records.

The impact of the proposed model is significant, as it provides a more lightweight and efficient approach for accurately detecting AD from MRI images. Unlike previous models that required deeper layers and more complex processing, the proposed model consists of only seven layers and can be employed in real-time applications with high detection performance. The end-to-end nature of the model also eliminates the need for traditional methods such as feature extraction and classification, making it a more streamlined and robust solution. By combining and reviewing all previous methods used on the same publicly available Kaggle dataset, this paper provides a comprehensive approach for researchers to work on a dataset with new challenges. Overall, the proposed model has the potential to improve the accuracy of AD detection and help in the early diagnosis, which can lead to earlier treatment and better management of the disease. In addition, the proposed model’s ability to work on both binary classification and multi-classification tasks with high accuracy makes it a useful tool for clinicians and researchers working in the field of AD.

## 2. Related Work

In recent years, techniques employing deep learning to diagnose AD have gained prominence [8,9,10,11,12,13,14,15,16]. Deep learning is a type of machine learning that is particularly well suited for the analysis of complex medical images, as it has the ability to automatically learn and extract features from large datasets. In addition to medical applications, deep learning is used in other applications [30,31,32]. For example, Darehnaei et al. [30] presented an approach for multiple vehicle detection in UAV images using swarm intelligence ensemble deep transfer learning (SI-EDTL). The presented method has the potential to enhance the effectiveness of various applications, such as surveillance and disaster response. A number of studies have explored the use of deep learning for AD detection using various imaging modalities, including structural MRI, functional MRI, PET, and amyloid imaging. However, in this paper, we focused on the methods that used the same dataset of MRI images. These studies have demonstrated the potential of deep learning to accurately classify the AD disease.

Several different approaches have been used to develop deep learning models for AD diagnosis using MRI images. Menagadevi et al. [8] developed a computer-aided diagnosis system for detecting AD based on a combination of a deep learning model with traditional classification methods. They first start with preprocessing stages on the input MRI images to enhance the images. After that, they perform segmentation on the preprocessed images to obtain the region of interest. Then, they extract the features using the presented multiscale pooling residual autoencoder model. Finally, they used separate classifiers such as K-Nearest Neighbor (KNN) and Extreme Learning Machine (ELM) for final classification. They obtained an overall accuracy of 96.88% using the KNN classifier and 98.97% using the ELM classifier for the binary classification task. However, the study focused on only one imaging modality, MRI, and used relatively small datasets. Murugan et al. [9] introduced a deep learning modality called “DEMNET” for diagnosing AD from MRI images. They used several image processing techniques, such as preprocessing, oversampling, and splitting the input data. After that, they fed the split data to the presented deep model for feature extraction and classification. They obtained an overall accuracy of 95.23% for the multi-classification task. However, similar to Menagadevi et al. [8], the study only focused on MRI imaging and used small datasets. Loddo et al. [10] presented a fully automatic model based on ensemble deep learning approaches for diagnosing AD from MRI images. They employed three pretrained deep models: AlexNet, ResNet 101, and InceptionResNetV2. After that, they used an average strategy to generate the ensemble output. They obtained the best accuracy of 96.57% for the binary classification task and an accuracy of 97.7% for the multi-classification task. However, the study did not use any image preprocessing techniques, and the ensemble approach may not always improve the performance of deep learning models. Sharma et al. [11] presented a hybrid modality called “HTLML” based on AI approaches for the detection of AD from MRI images. They perform the first preprocessing stage on the input MRI images. After that, they fed these preprocessed images in parallel into two pretrained models, such as DenseNet201 and DenseNet121. Then, they perform classification using separate classifiers for each pretrained model. Finally, they combine the output for each classifier using the voting strategy to obtain the final decision. They obtained an overall accuracy of 91.75% for the multi-classification task. However, the study did not employ any data augmentation techniques and used relatively small datasets. Hazarika et al. [33] presented an approach for the classification of AD using deep neural networks and MRI. The approach involves preprocessing the MRI scans, extracting features from segmented brain images using a combination of 2D and 3D CNNs, and classifying the scans into AD and non-AD using a fully connected neural network. The authors achieved promising results with an accuracy of 95.34%, a sensitivity of 96% and a specificity of 94.67%. The presented method has the potential to significantly improve the early detection and treatment of AD. However, further validation on larger and more diverse datasets is necessary to assess its generalizability and robustness.

Another hybrid model based on deep learning and traditional classifiers was presented by Mohammed et al. [12] for the early diagnosis of AD from MRI images. The authors first enhanced the input MRI images using several preprocessing techniques. After that, they fed the preprocessed images to the presented deep model, which is a convolutional neural network (CNN) model for extracting the features. Finally, these features are fed to a separate classifier, such as a support vector machine (SVM), for final classification. They worked on a multi-classification task and obtained an overall accuracy of 94.80%. However, the use of a traditional classifier may limit the performance of the model. Balasundaram et al. [13] used the ResNet50 pretrained model for the diagnosis of AD from MRI images. They used preprocessing techniques such as resizing and thresholding on the input images. After that, they fed these images to the presented pretrained model for final classification. They obtained an overall accuracy of 94.1% on the multi-classification task. However, the study did not employ any data augmentation techniques, and the use of a single pretrained model may limit the performance of the model. Bangyal et al. [14] applied deep learning techniques to MRI images to detect AD. A comparative analysis between them proves that deep learning approaches can detect AD better than traditional machine learning approaches. They finally obtained an overall accuracy of 94.63% using deep learning approaches on a multi-classification task from MRI images. However, the study did not employ any data augmentation techniques and used relatively small datasets. Ahmed et al. [15] presented a classification method called “DAD-Net” using an optimized neural network for the early diagnosis of AD. They split the data and performed preprocessing techniques on the input MRI images. After that, they fed these images to the presented deep classification model for extracting features and final classification. They obtained an overall accuracy of 90% for the multi-classification task. Tuvshinjargal and Hwang [16] presented a combination model between the VGG-C transform and CNN for the prediction of AD from MRI images. They use Z-score scaling to preprocess the input images and quantize pixel intensity. After that, they fed these images to the VGG pretrained model for final prediction. They obtained an overall testing accuracy of 77.46 when working on a multi-classification task. However, the study used a relatively simple deep learning model and achieved lower performance compared to the other studies. Balaji et al. [34] presented a hybridized deep learning approach for detecting AD using MRI images. The authors combine a CNN and a long short-term memory (LSTM) network to learn spatial and temporal features from MRI scans. The authors report an accuracy of 98.50% in classifying MRI scans into AD or normal cases using the presented hybridized deep learning approach. However, the study requires a large amount of data to learn complex features and patterns accurately. In addition, the use of this combination can be computationally expensive, which may limit the scalability of the model. Hu et al. [22] introduced a deep learning model for a short-term longitudinal study of MCI using brain structural MRI (sMRI) as the main biomarker. The VGG-TSwinformer model combines a VGG-16-based CNN and Transformer to extract and encode features from longitudinal sMRI images, and it uses sliding-window and temporal attention mechanisms to integrate local and distant spatial features for MCI progression prediction. They obtained an accuracy of 77.20% for the binary classification task. The study still has some limitations, such as not mining 2D local features inside slices, not adopting an effective feature fusion method for axial, coronal and sagittal plane slices, and not taking full advantage of available cross-sectional biomarkers.

The summary of all previous methods is shown in Table 1, along with the disadvantages of each method. In this study, we present a novel lightweight CNN model that overcomes the previous limitations for all related work with higher performance in both binary and multi-classification tasks. Our model and the dataset we used are discussed in detail in the following section.

## 3. Dataset and Methodology

This section discusses in detail the used dataset and provides visual examples of the data. In addition, all stages of the proposed model with all hyperparameters are also discussed in this section.

### 3.1. Dataset

**Figure 1:**
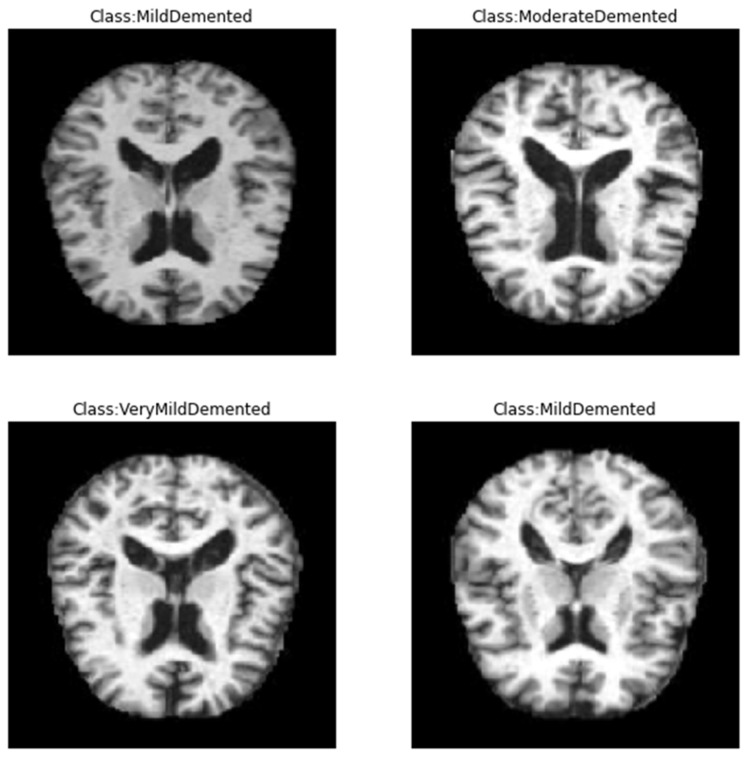
Sample of the data from Kaggle database [29].

**Figure 2:**
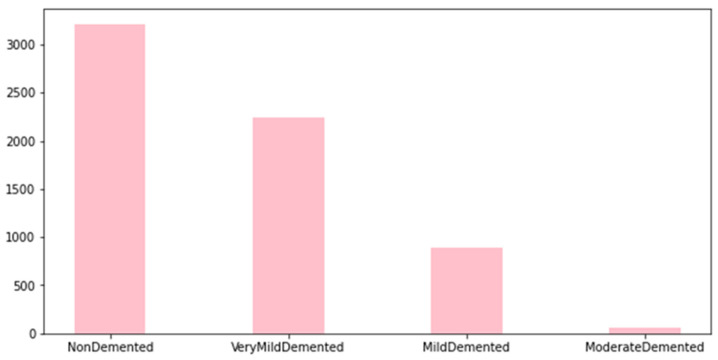
Statistics of Kaggle dataset [29].

In this study, we employed the Alzheimer’s dataset [29], which is a hand-collected dataset consisting of MRI images that have been verified and labeled by experts. The data includes four different classes of images: Mild Demented, Moderate Demented, Non-Demented, and Very Mild Demented. These images can be used to train and test deep learning models aimed at accurately predicting the stage of AD. The dataset provides an opportunity for researchers and practitioners to develop algorithms that can accurately diagnose AD and aid in the development of effective treatments. With the growing global burden of AD, this dataset could play an important role in advancing our understanding of the disease and improving patient outcomes. The dataset is available on Kaggle and can be easily accessed, unlike other datasets, which are difficult to access. By making this dataset publicly available, the creators aim to encourage more research in the field and support the development of better algorithms for the diagnosis and treatment of AD. We employed this dataset as it is totally free, available with different classes, and small in size on a hard disk, unlike other common datasets in this field. Figure 1 shows samples of the dataset for the different classes. Table 2 shows the distribution of the records in the dataset, and Figure 2 shows the statistics of this dataset. Furthermore, a comparison between this dataset and other common datasets in this field is shown in Table 3.

**Figure 3:**
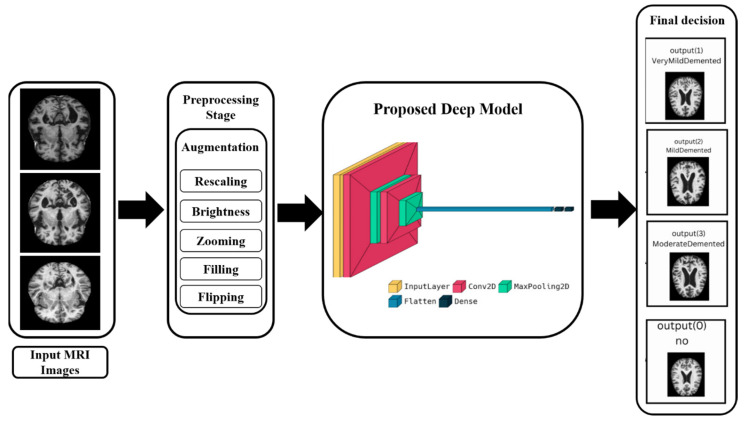
Block diagram of all stages of our method.

The difficulty with this dataset is that when working on a multi-classification task, we can see that the class Moderate Demented has a very low number of images compared to other classes, which causes false positives and affects the final results. We solve this problem by using a data augmentation technique to increase the number of images in this class while also addressing the imbalance issue. Figure 3 shows the general block diagram of our method, which is discussed in detail in the next section.

### 3.2. Preprocessing Stage

In this stage, we used the ImageDataGenerator class from the Keras library, where various image augmentation techniques can be applied to the input data, generating a new set of augmented images that can be used for training. The specific augmentation techniques applied in this paper include the rescaling of pixel values, brightness adjustments, zoom changes, filling in new pixels created by augmentation with a constant value, and random horizontal flipping of images. These techniques aim to artificially increase the size of the training dataset as well as make the model more robust to variations in the input data. Once the ImageDataGenerator instance has been defined, the input image data can be fed into our deep model, and the resulting augmented data can be used for training a deep learning model. This step is important in ensuring that the model is able to generalize well to new or unseen data.

### 3.3. Proposed Deep Model for Binary-Classification

**Figure 4:**
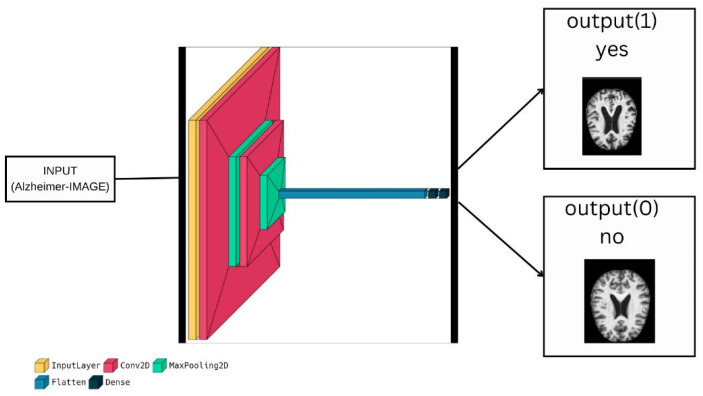
Model visualization to binary classification task.

**Figure 5:**
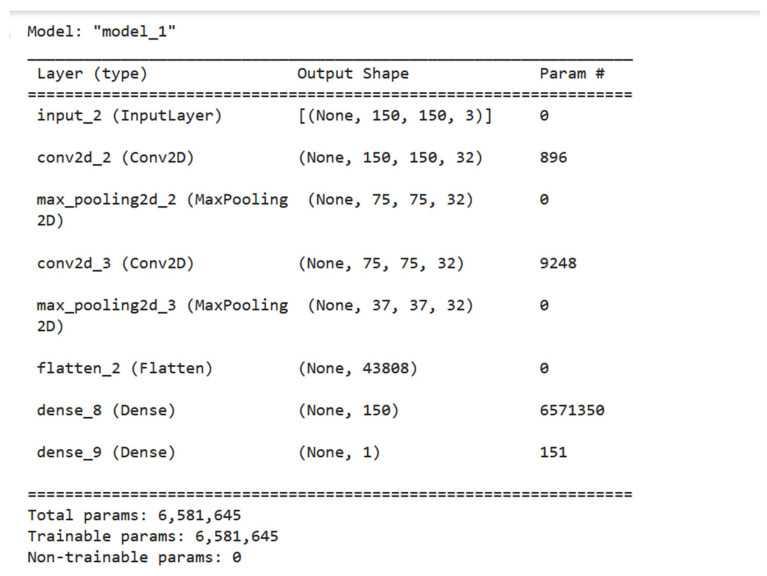
Summary of the proposed model for binary classification.

The proposed deep learning model was created using Keras, which is a high-level neural network API built on top of TensorFlow [37]. The model is designed for binary classification, with the goal of predicting whether an input image is of a certain class or not. Figure 4 shows the visualization of our model for a binary classification task. The model starts with an input layer that takes in an image of size 150 × 150 × 3 (height × width × depth), which represents a color image with 3 channels (red, green, and blue). The image is then processed through a series of convolutional layers (Conv2D) and pooling layers (MaxPooling2D) to extract features from the image. The convolutional layers apply filters to the input image, and the pooling layers down-sample the feature maps produced by the convolutional layers. The features are then flattened and passed through two dense layers (Dense), which use the activation function ‘ReLU’ to perform non-linear transformations on the features. Finally, the output layer uses the sigmoid activation function, which maps the input to a probability-like output between 0 and 1, to produce the final prediction. The binary cross-entropy loss function and the ‘Adam’ optimizer are used to construct the model [38], and they are trained on the training data using the fit method. The training results show a loss of 0.061 and an accuracy of 0.993, indicating that the model is able to make accurate predictions based on the training data. The summary of the proposed model for binary classification is shown in Figure 5. From the summary, we can see that the model is lightweight, as it consists of only 7 layers with total parameters of 6581645. As a result, we reduce the complexity of the method and decrease the processing time.

### 3.4. Proposed Deep Model for Multi-Classification

**Figure 6:**
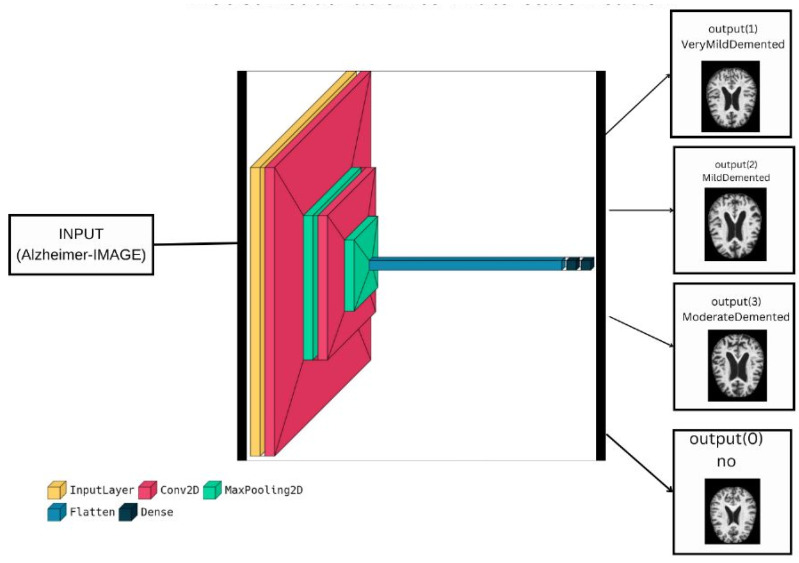
Model visualization to multi-classification task.

**Figure 7:**
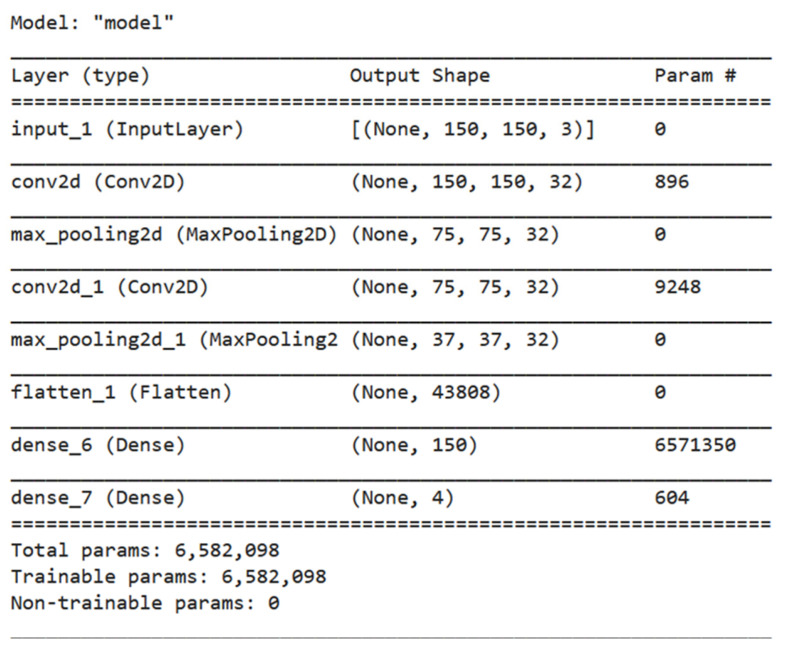
Summary of the proposed model for multi-classification.

This model takes as input images with shape (150, 150, 3), which means that each image is 150 × 150 pixels and has 3 color channels (red, green, blue). The model then applies a series of Conv2D and MaxPooling2D layers to reduce the spatial dimensions of the image and extract meaningful features from it. The extracted features are then flattened and passed through two dense layers with activation functions ‘ReLU’ and ‘SoftMax’. The ‘SoftMax’ activation function provides the final probability scores for each class in the classification task. The model is then compiled with an optimizer ‘Adam’ and a categorical cross-entropy loss function. The model is trained on the training data with 100 epochs and evaluated on the validation data, resulting in an accuracy of 96%. Figure 6 shows the visualization of our model for multi-classification tasks. The summary of the proposed model for multi-classification is shown in Figure 7. From the summary, we can show that the model is lightweight as it consists of only 7 layers with total parameters of 6582098, which is almost the same number as the first model. As a result, we reduce the complexity of the method and decrease the processing time.

The pseudocode of our algorithm is shown in Algorithm 1. The proposed deep learning model for binary and multi-classification tasks is based on a lightweight architecture that can be trained efficiently on large datasets. The use of data augmentation techniques helps to improve the generalization performance of the model, making it more robust to variations in the input data. The model achieves high accuracy on the validation dataset, demonstrating its effectiveness in performing binary and multi-classification tasks on image datasets.

Compared to existing methods, the proposed model is simpler in terms of the number of layers and parameters, which results in faster training times and lower computational resources. Additionally, the use of data augmentation techniques further improves the model’s performance without requiring additional data or computational resources. The proposed model can be used in a variety of applications, including medical image analysis, autonomous driving, and object recognition in robotics.
**Algorithm 1:** The proposed algorithm (Summary of the proposed model)1. BEGIN2. INPUT: dataset_directory, training_percentage, image_augmentation_parameters, model_parameters, optimizer, loss_function, performance_metrics.3. Load input dataset from dataset_directory4. Split the dataset into training set and validation set with training_percentage5. Instantiate an ImageDataGenerator object with image_augmentation_parameters6.     IF model_parameters is a pre-trained model THEN7.      Load pre-trained model8.     ELSE9.       Define a deep learning model using Keras with model_parameters10.      ENDIF11. Compile the model using optimizer and loss_function12. Train the model on the training set for a number of epochs with the compiled model and ImageDataGenerator object13.      FOR each epoch in the training process DO14.         Evaluate the model on the validation set using performance_metrics15.           IF the validation accuracy is not improving THEN16.       Reduce learning rate17.      ENDIF18.      ENDFOR19. Test the final model on a separate test set to evaluate its generalization performance using performance_metrics20. OUTPUT: the performance_metrics of the proposed method and existing methods for comparison21. END

**Figure 8:**
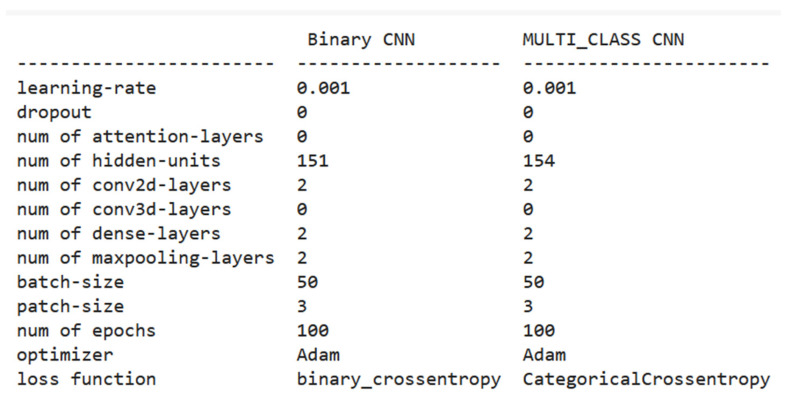
Summary of the used hyperparameters for our models.

The hyperparameters for both models are almost the same except for the number of hidden layers and the used loss function. In binary classification (the first model), we employed 0.001 for the learning rate with 151 units in hidden layers and a batch size of 50. This model is finished after 100 epochs with the loss function binary_crossentropy function and ‘Adam’ optimizer. In multi-classification (the second model), we employed 0.001 for the learning rate with 154 units in hidden layers and batch size of 50. This model is finished after 100 epochs with the loss function, CategoricalCrossentropy function, and the Adam optimizer. The summary of all parameters for both models is shown in Figure 8.

## 4. Results and Discussion

Multiple experiments are carried out using the proposed deep model from the Kaggle dataset to train the aforementioned AD deep model. For the training and testing, a standard approach of cross-validation (10-CV) [39] was used for a fair and reliable evaluation of the proposed AD detection model. The proposed approach is implemented on a computer with a GPU (specifically, an NVIDIA Tesla T4 GPU) and 14 GB DDR4 RAM. Keras, a Python-based library, is used for the implementation. For training the neural network, the ‘Adam’ optimizer is applied, and the binary cross-entropy class is used as the loss function for model 1, and CategoricalCrossentropy is used as a loss function for model 2. In this study, four evaluation measures were used: *Accuracy*, *Precision*, *Recall*, and *F1-score*. These metrics are defined as follows:

(1) $$Accuracy=\frac{T_{P}+T_{N}}{T_{P}+T_{N}+F_{P}+F_{N}}$$

(2) $$Precision=\frac{T_{P}}{T_{P}+F_{P}}$$

(3) $$Recall=\frac{T_{P}}{T_{P}+F_{N}}$$

(4) $$F1\text{-}score=2\times \frac{Precision\times Recall}{Precision+Recall}$$

where *T_P_* denotes true positives, *F_P_* denotes false positives, *T_N_* denotes true negatives and *F_N_* denotes false negatives.

### 4.1. Experimental Analysis

Two experiments are evaluated in this paper based on the previous four metrics. The first experiment is based on the first model for a binary classification task, whereas the second experiment is based on the second model for multi-classification tasks. The following are the details of each experiment with the analysis:

A. The first experiment

**Figure 9:**
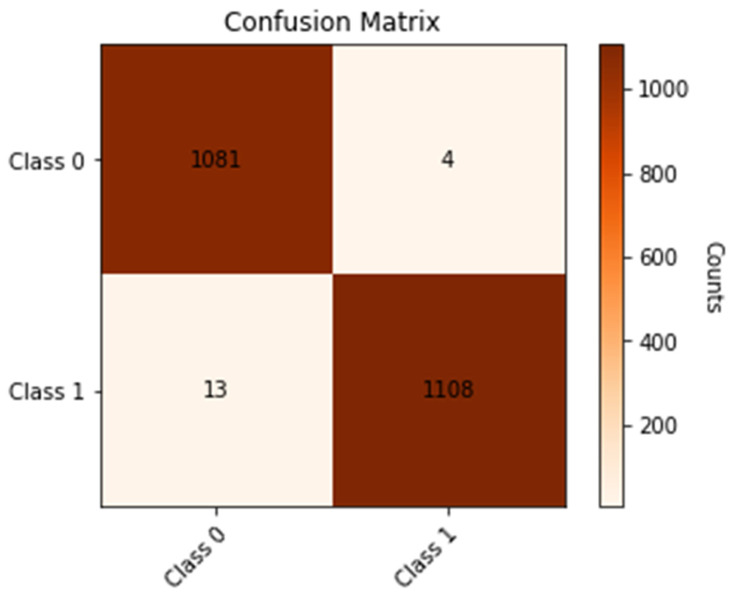
Confusion matrix of the proposed method to detect AD for binary classification tasks.

We classified input MRI images into two groups using the proposed deep model for a binary challenge (AD or Normal). Figure 9 depicts the confusion matrix of the proposed approach for detecting AD, in which class 0 represents normal instances and class 1 represents AD patients.

**Figure 10:**
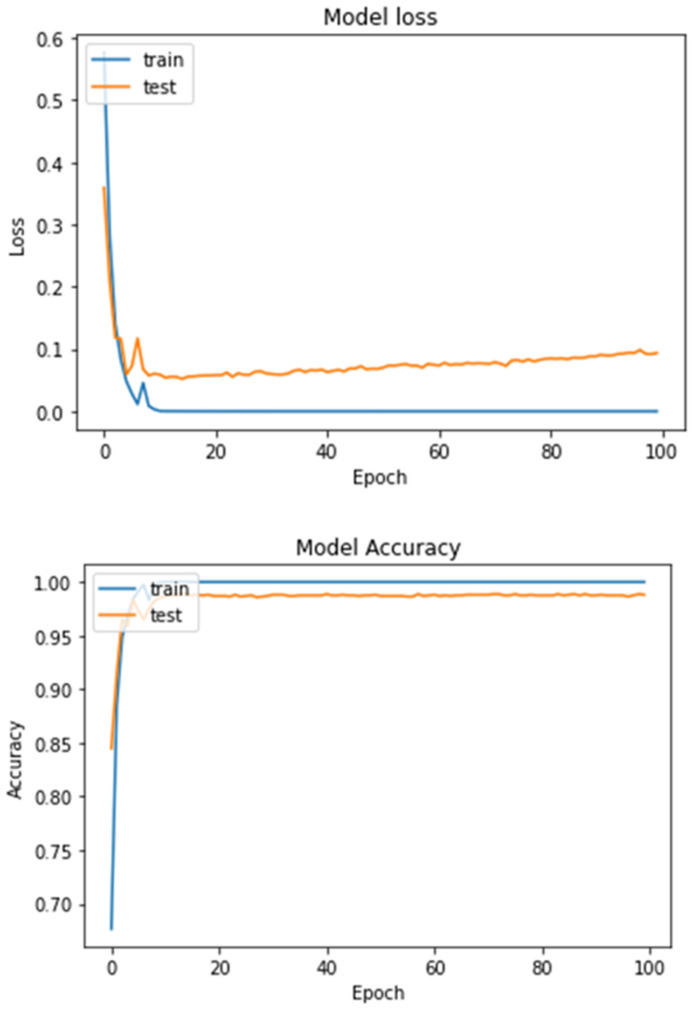
Loss curves (**upper**) and accuracy curves (**lower**) for the training and testing data for the proposed model for binary classification task.

From the previous confusion matrix shown in Figure 10, we can show that the number of normal MRI images that were detected as normal is 1081, and 0.3% of the normal cases are detected as AD cases. In addition, we can find that 98% of the AD cases are correctly detected as AD and that 13 MRI images are detected wrongly as normal cases. The performance of the proposed model is shown in Table 4.

From the performance table, we can observe that the overall accuracy of the proposed model is 99.22%, the overall precision and recall are both 99.22%, and the overall F1-score is 99.21%. Figure 10 shows the loss curves (left) and accuracy curves (right) for the training and testing data of the proposed model. From the curves, we can see that the accuracy of the model is stable after 20 epochs and has not changed, and the loss of training data are stable after 15 epochs. For the testing data, the loss is slightly increased after 20 epochs and totally stable after 80 epochs.

B. The second experiment

**Figure 11:**
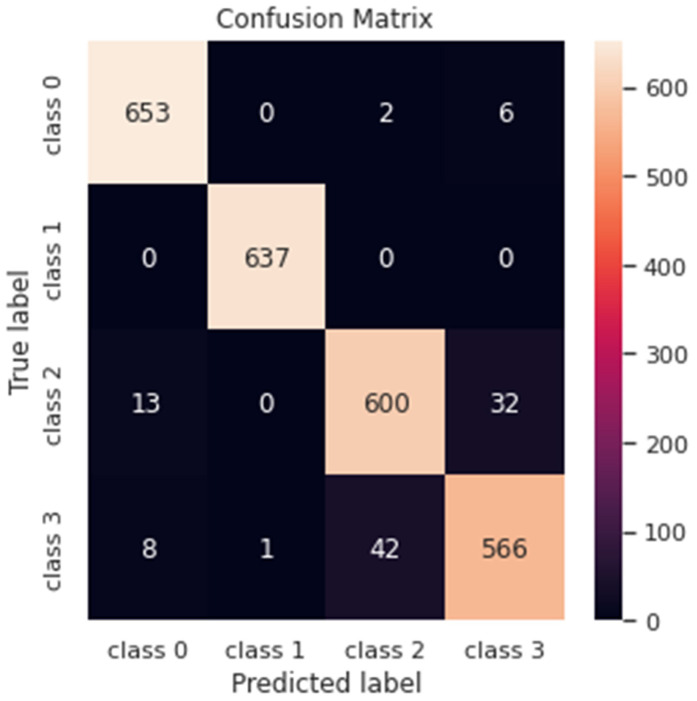
Confusion matrix of the proposed model for multi-classification task.

In this case, we used the proposed deep model for multi-classification to categorize the input MRI images into four categories (Mild Demented, Moderately Demented, Non-Demented, and Very Mild Demented). Figure 11 shows the confusion matrix of the proposed method to detect demented cases, where Class 0 refers to Non-Demented cases, Class 1 refers to Very Mild Demented cases, Class 2 refers to Mild Demented cases and Class 3 refers to Moderate Demented cases.

According to the previous confusion matrix in Figure 11, 653 Non-Demented cases were correctly detected as Non-Demented; 2 MRI images of Non-Demented cases were incorrectly detected as Mild Demented cases, and 6 MRI images were correctly detected as Moderately Demented cases. We can also find that 100% of the Very Mild Demented cases are correctly detected as Very Mild Demented cases. In addition, we can observe that 93% of the Mild Demented cases are correctly detected as Mild Demented, 4.9% of the images are wrongly detected as Moderate Demented, and 2.1% are wrongly detected as Non-Demented cases. Finally, we can also observe from the confusion matrix that 91.7% of the Moderate Demented cases are correctly detected as Moderate Demented, 42 MRI images are wrongly detected as Mild Demented, 1.29% are wrongly detected as Non-Demented cases and 0.16% of the images are wrongly detected as Very Mild Demented cases. Table 5 shows the overall performance of the proposed model for multi-classification tasks.

**Figure 12:**
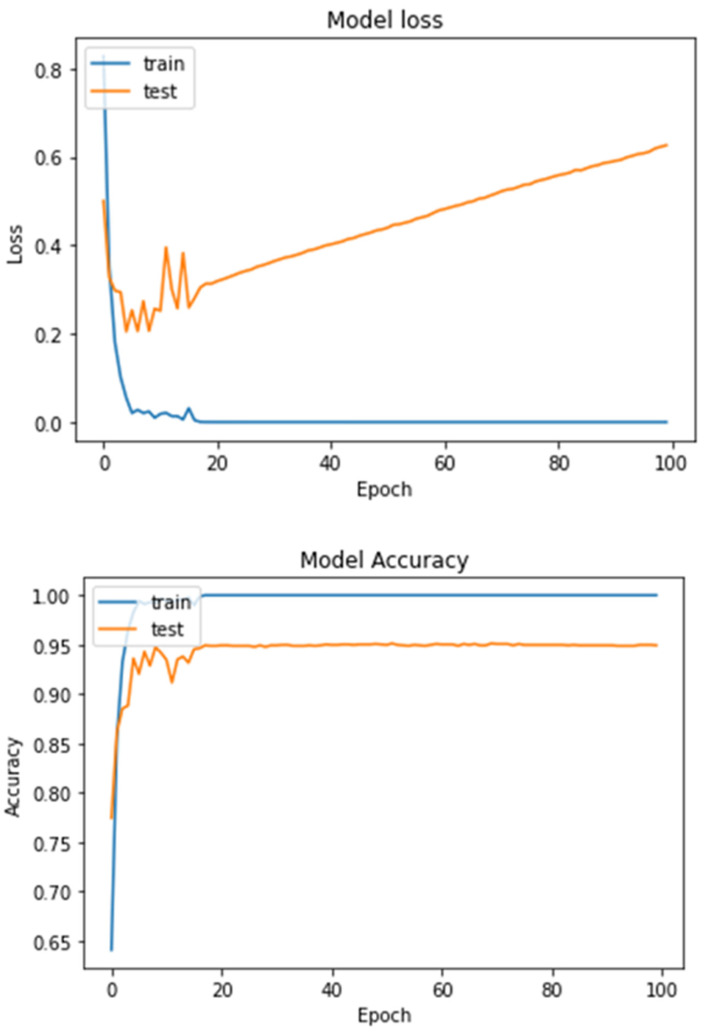
Loss curves (**upper**) and accuracy curves (**lower**) for the training and testing data for the proposed model for multi-classification task.

From the performance table, we can observe that the overall accuracy of the proposed model is 95.93%, the overall precision is 95.93%, the overall recall is 95.88%, and the overall F1-score is 95.90%. Figure 12 shows the loss curves (left) and accuracy curves (right) for the training and testing data of the proposed model. From the curves, we can see that the accuracy of the model is stable after 30 epochs and has not changed, and the loss of training data is stable after 20 epochs. For the testing data, the loss is slightly increased after 20 epochs and totally stable after 90 epochs.

### 4.2. Comparison with Other State-of-the-Art Deep Models

In this section, we will conduct a comprehensive comparison between a state-of-the-art deep learning model for our model for AD detection and other prominent models that have been developed for the same task. The comparison will be based on the same dataset and provide insights into the relative performance of the different models. Table 6 compares our model with various cutting-edge techniques for binary classification problems. Table 7 contrasts our model for multi-classification tasks with previous state-of-the-art deep methods on the same dataset. Table 8 shows a comparison of our method with recent papers using several datasets.

From the previous tables, it is evident that the proposed model is more robust than earlier models, which attained the greatest accuracy among other approaches for both binary classification and multiclassification tasks. Menagadevi et al. [8] obtained good accuracy in both tasks, but our method was still better. Furthermore, they used a very complex model with separate classifiers, which, unlike our lightweight model, is unsuitable for real-world applications. Using a pretrained model for a multi-classification task gives better accuracy than a binary classification task, as in [11,12,13,16]. The normal CNN model as in [9,14,15] obtains acceptable accuracy but is still worse than our model and the combination of deep learning with traditional classification as in [8,10]. Loddo et al. [10] obtained very low accuracy for binary tasks compared with our model and better than our method for multi-classification tasks. However, the model they used has a higher time and cost complexity than ours, making it unsuitable for real-time applications. Finally, we can confirm that, we proposed a robust deep learning model that is more robust than other recent deep learning models for AD detection.

### 4.3. Computational Complexity

Complexity analysis is an important aspect of evaluating the performance of any algorithm, and it is especially critical when working with large datasets. In this section, we will perform a complexity analysis of our lightweight deep learning model for the accurate detection of AD using MRI data. The proposed deep learning model uses a reduced number of parameters and layers compared to traditional CNNs, making it computationally more efficient. The training complexity of our model is O(m ∗ n), where *m* is the number of training examples and *n* is the number of pixels in each input image. The reduced number of parameters and layers in our model allows for faster convergence and more efficient training compared to traditional CNNs.

Based on the above analysis, we can see that the proposed lightweight deep learning model for the accurate detection of AD using MRI data is computationally efficient in terms of time and training complexity. This makes it suitable for deployment on limited hardware resources, such as edge devices or mobile devices, without the need for specialized hardware. The reduced complexity also allows for faster convergence and more efficient training, leading to improved performance and accuracy in detecting AD. Table 9 shows the computational complexity required to accomplish the performance accuracy for training the proposed deep model.

## 5. Conclusions

This study aims to evaluate the performance of deep learning models in detecting and classifying AD using MRI images. The results obtained in the binary classification task, with an accuracy of 99.30%, and in the four-class classification task, with an accuracy of 95.96%, demonstrate the potential of deep learning models for accurately detecting and differentiating between the different stages of AD. The use of image data with a shape of 150 × 150 × 3, as well as image augmentation techniques and a SoftMax activation function with a dense four-output layer, were found to be critical factors in achieving these results.

Our study contributes to the growing body of literature on the use of deep learning models for AD detection and classification. Specifically, our study demonstrates the potential of using MRI images and deep learning models to accurately detect and classify AD, which has important implications for early diagnosis and treatment. Moreover, the findings of our study provide a foundation for future research in this area.

However, our study has some limitations that should be considered. First, the dataset used in this study is relatively small, and it may not be representative of the entire population. Second, our study only considered a single modality (MRI images), and future studies could explore the use of other imaging modalities (e.g., PET scans) in combination with deep learning models. Finally, the generalizability of our findings may be limited by the specific deep learning architecture and hyperparameters used in this study.

Future work in this area could focus on addressing the limitations of our study as well as exploring the use of deep learning models in other areas of medical imaging. Additionally, the development of more explainable deep learning models that can provide insights into the underlying biological mechanisms of AD could further advance our understanding of this disease.

## Figures and Tables

**Table 1:** Summary of previous works for AD detection based on deep learning.

Authors and Reference	Methodology	Disadvantages
Menagadevi et al. [8] (2023)	Pooling residual autoencoder + ELM	- Complex model - Classifier-dependent method - Require more time for training - Not suitable for real applications
Murugan et al. [9] (2021)	Preprocessing + CNN + RMS	- High computational complexity - Overfitting problem - A problem in model convergence. - Low performance on big data
Loddo et al. [10] (2022)	Pretrained models + Ensemble classifier	- Less interpretable - Time and cost complexity - Not suitable for real applications - Low accuracy on big and small data
Sharma et al. [11] (2022)	Pretrained models + SVM	- Low accuracy with big data - Perform poorly in imbalanced datasets - Overfitting problem - Classifier dependent method
Mohammed et al. [12] (2021)	Pretrained models + SVM	
Balasundaram et al. [13] (2023)	Segmentation + Pretrained models	- Overfitting problem - Takes large number of resources (time and computation power) - Not suitable for real applications - Low performance on big data
Bangyal et al. [14] (2022)	CNN	- Exploding gradient - Problem with imbalanced datasets - Overfitting problem - Complex model
Ahmed et al. [15] (2022)	Preprocessing + CNN + optimization method	- Obtained low accuracy - Complex model - Not suitable for real applications
Tuvshinjargal and Hwang [16] (2022)	Preprocessing + pretrained model	- Overfitting problem - High computational complexity - Low accuracy on big data - Not suitable for real applications - Perform poorly in imbalanced datasets
Hazarika et al. [33] (2023)	Preprocessing + 2D CNN and 3D CNN	- Obtained low accuracy for binary classification task - Low accuracy with big data - Not robust
Balaji et al. [34] (2023)	3D CNN + LSTM	- Difficult to understand how the model is making its predictions - Computationally expensive - Require a large amount of data to learn complex features accurately
Hu et al. [22] (2023)	Pretrained model + CNN	- Not adopting an effective feature fusion method for axial - Obtained very low accuracy for binary classification task - Not robust

**Table 2:** Distribution of the records in the dataset used in this work.

	Mild Demented	Moderate Demented	Non-Demented	Very Mild Demented
Train	717	52	2560	1792
Test	179	12	640	448

**Table 3:** Comparison between Kaggle dataset and other common datasets in this field.

Dataset	Number of Subjects	Number of Classes	Size on Desktop	Availability
ADNI [35]	822	3	5 GB	Need Access
OASIS [36]	416	2	1.5 GB	Need Access
Kaggle [29]	6400	4	32 MB	Publicly Available

**Table 4:** The performance of the proposed model for binary classification task.

	Precision	Recall	F1-Score
0	98.81%	99.63%	99.21%
1	99.63%	98.81%	99.21%
Accuracy	99.22%		
Macro Avg	99.22%		
Weighted Avg	99.22%		

**Table 5:** The performance of the proposed model for multi-classification task.

	Precision	Recall	F1-Score
0	96.88%	98.78%	97.82%
1	100%	100%	100%
2	93.16%	93.02%	93.08%
3	93.70%	91.73%	92.70%
Accuracy	95.93%		
Macro Avg	95.93%		
Weighted Avg	95.93%		

**Table 6:** Comparison between the proposed model with other previous model for binary classification task.

Reference/Author	Methodology	Performance
Menagadevi et al. [8] (2023)	Deep residual autoencoder + ELM	Accuracy = 98.97%
Loddo et al. [10] (2022)	Deep-Ensemble	Accuracy = 96.57% Sensitivity = 96.57% Specificity = 98.28% F1-score = 96.57%
Tuvshinjargal and Hwang [16] (2022)	Pretrained model	Accuracy = 0.774 Precision = 0.774 Recall = 0.785 F1-score = 0.779
Proposed (2023)	Lightweight deep model	Accuracy = 99.22% Precision = 99.22% Recall = 99.22% F1-score = 99.21%

**Table 7:** Comparison between the proposed model with other previous model for multi-classification task.

Reference/Author	Methodology	Performance
Murugan et al. [9] (2021)	CNN	Accuracy = 95.23% Precision = 96% Recall = 95% F1-score = 95.27%
Loddo et al. [10] (2022)	Deep ensemble	Accuracy = 97.71% Sensitivity = 96.67% Specificity = 98.22%
Sharma et al. [11] (2022)	Pretrained deep models	Accuracy = 91.75%
Mohammed et al. [12] (2021)	Pretrained deep model + SVM	Accuracy = 94.80% Sensitivity = 93% Specificity = 97.75%
Balasundaram et al. [13] (2023)	Pretrained deep models	Accuracy = 94.10% Precision = 96.50% Recall = 94.75% F1-score = 95.5%
Bangyal et al. [14] (2022)	CNN	Accuracy = 94.63% Precision = 94.75% Recall = 94.75% F1-score = 94.50%
Ahmed et al. [15] (2022)	CNN	Accuracy = 90% Precision = 91.34% Recall = 87.34% F1-score = 88.09%
Proposed (2023)	Lightweight deep model	Accuracy = 95.93% Precision = 95.93% Recall = 95.88% F1-score = 95.90%

**Table 8:** Comparison between the proposed model with other recent models on several datasets.

Reference/Authors	Methodology	Database	Classification Task	Performance
Hazarika et al. [33] (2023)	Hybrid pretrained models	ADNI [35]	Multi-classification	Accuracy = 88% Precision = 92% Recall = 90% F1-score = 91%
Balaji et al. [34] (2023)	CNN + LSTM	Kaggle [29]	Binary classification	Accuracy = 98.50% Precision = 94.80% Recall = 98%
Hu et al. [22] (2023)	Pretrain model + Transformer	ADNI [35]	Multi-classification	Accuracy = 77.20% Sensitivity = 79.97% Specificity = 71.59%
Sethuraman et al. [40] (2023)	Hybrid pretrained models	ADNI [35]	Binary classification	Accuracy = 96.61% Sensitivity = 94.34% Specificity = 94.96%
Shojaei et al. [41] (2023)	3D CNN	ADNI [35]	Binary classification	Accuracy = 96.60%
EL-Geneedy et al. [23] (2023)	CNN	OASIS [36]	Binary classification	Accuracy = 99.68%
Proposed	Lightweight CNN	Kaggle [29]	Binary classification and multi-classification	For binary classification: Accuracy = 99.22% Precision = 99.22% Recall = 99.22% F1-score = 99.21% For multi-classification: Accuracy = 95.93% Precision = 95.93% Recall = 95.88% F1-score = 95.90%

**Table 9:** Computational complexity required to accomplish the performance accuracy for training the proposed deep model.

Steps	Complexity in Big-O Notation
Handling the unbalanced problem of the dataset	Depends on the size of the data, which is not more than O(n)
The inner steps (number of parameters and layers)	O(m) ∗ O(1) Where O(1) is the time required for each layer of the proposed model
Training the deep learning model	O(m ∗ n), where m is the number of training examples and n is the number of pixels in each input image
The total big-O	O(m ∗ n)

## Data Availability

All data will be available upon reasonable request from the corresponding authors.
